# Implementation outcomes of the national scale up of chlorhexidine cord cleansing in Bangladesh’s public health system

**DOI:** 10.7189/jogh.09.020410

**Published:** 2019-12

**Authors:** Jennifer A Callaghan-Koru, Marufa Khan, Munia Islam, Ardy Sowe, Jahurul Islam, Sk Masum Billah, Imteaz Ibne Mannan, Joby George, The Bangladesh Chlorhexidine Scale Up Study Group

**Affiliations:** 1 Department of Sociology, Anthropology, and Health Administration and Policy, University of Maryland, Baltimore County, Baltimore, Maryland, USA; 2 Save the Children International, Bangladesh, Dhaka, Bangladesh; 3 MaMoni Health Systems Strenghtening Project; 4 Ministry of Health and Family Welfare, Government of Bangladesh, Dhaka, Bangladesh; 5 Maternal and Child Division, icddr,b, Dhaka, Bangladesh; 6 Jhpiego (formerly, Save the Children, Bangladesh), Kabul, Afghanistan

## Abstract

**Background:**

Chlorhexidine (CHX) cleansing of the umbilical cord stump is an evidence-based intervention that reduces newborn infections and is recommended for high-mortality settings. Bangladesh is one of the first countries to adopt and scale up CHX nationally. This study evaluates the implementation outcomes for the CHX scale up in Bangladesh and identifies and describes key milestones and processes for the scale up.

**Methods:**

We adapted the RE-AIM framework for this study, incorporating the WHO/ExpandNet model of Scale Up. Adoption and incorporation milestones were assessed through program documents and interviews with national stakeholders (n = 25). Provider training records served as a measure of reach. Implementation was assessed through a survey of readiness to provide CHX at public facilities (n = 4479) and routine data on the proportion of all live births at public facilities (n = 813 607) that received CHX from December 2016 to November 2017. Six rounds of a rolling household survey with recently-delivered women in four districts (n = 6000 to 8000 per round) measured the effectiveness and maintenance of the scale up in increasing population-level coverage of CHX in those districts.

**Results:**

More than 80 000 providers, supervisors, and managers across all 64 districts received a half-day training on CHX and essential newborn care between July 2015 and September 2016. Seventy-four percent of facilities had at least 70% of maternal and newborn health providers with CHX training, while only 46% had CHX in stock on the day of the assessment. The provision of CHX to newborns delivered at facilities steadily increased from 15 059 newborns (24%) in December 2016 to 71 704 (72%) in November 2017. In the final household survey of four districts, 33% of newborns were reported to receive CHX, and babies delivered at public facilities had 5.04 times greater odds (95% CI = 4.45, 5.72) of receiving CHX than those delivered at home.

**Conclusions:**

The scale up of CHX in Bangladesh achieved sustained national implementation in public health facilities. Institutionalization barriers, such as changes to supply logistics systems, had to be addressed before expansion was achieved. For greater public health impact, implementation must reach deliveries that take place at home and in the private sector.

In global maternal and newborn health, there is a well-documented gap in coverage of known evidence-based practices [1-3] and a recognized need for implementation research that facilitates improved delivery of interventions to the mothers and children who need them [4-6]. The scale up of new evidence-based practices is an area of increasing implementation research focus in global health [7-9]. The WHO/ExpandNet model defines scale up as the “deliberate efforts to increase the impact of successfully tested health innovations so as to benefit more people and to foster policy and program development on a lasting basis” [10]. Although multiple frameworks have been developed to guide or understand scale up [7-12], there remains a great need for empirical studies of scale up efforts that identify determinants of successful scale up in low-income countries [6,13].

Chlorhexidine (CHX) cleansing of the umbilical cord stump is a new evidence-based newborn health intervention, recently endorsed by the WHO for high-mortality settings, which provides an opportunity to study scale up in low-income country contexts. Multiple trials in Asia and Africa have demonstrated CHX to be effective in reducing neonatal infections and mortality [14]. Bangladesh, which hosted one of the CHX trials [15], adopted CHX within its national newborn health policy in 2013 [16], and in 2015 became one of the first countries to start national scale up of CHX.

The scale up effort in Bangladesh was informed by the WHO/ExpandNet Framework for Scale Up (ExpandNet) [17]. ExpandNet distinguishes between two distinct types of scale up – institutionalization, or “vertical” scale up and expansion, or “horizontal” spread of the evidence-based practice. Institutionalization involves adoption and incorporation of the practice into policies, plans, budgets, and health system structures, while expansion involves replication or extension of the practice to new locations or populations [18]. Although the ExpandNet framework has been increasingly used to guide and study geographically limited scale up of programs in low-income countries, particularly in the area of reproductive health [19-21], there has been limited systematic study of the nationwide scale up processes and outcomes, particularly related to the institutionalization dimension of scale up in ExpandNet’s model.

We conducted an evaluation of the CHX scale up in Bangladesh with a goal of providing lessons that can be incorporated into the scale up plans of other countries implementing CHX and similar interventions. The objectives of this study are: 1) to evaluate the implementation outcomes for the CHX scale up in Bangladesh; 2) to identify and describe key milestones and processes for institutionalization and expansion, to guide future efforts in other countries. A companion paper qualitatively identifies barriers and facilitators for the scale up of CHX.

## METHODS

### Setting and intervention

Bangladesh, a densely-populated low-income country in Southeast Asia, has made great progress in improving child health in recent decades and achieved its Millennium Development Goal 4 target of reducing the under-five mortality rate to 48 deaths per 1000 live births [22]. As overall child deaths declined, the proportion of child deaths that take place during the newborn period has steadily increased and is now 61% of under-five deaths [22]. Skilled delivery care, a critical intervention point for newborn survival, is low. In 2016, 47% of babies were delivered in a health facility, with 14% of deliveries taking place in public sector facilities, 29% in private sector facilities, and 4% in NGO-operated facilities [23].

Based on the evidence from community-based trials, the WHO included formal recommendations for CHX cleansing of the umbilical cord stump to reduce infection-related newborn deaths in high mortality settings in their 2013 guideline document, “Postnatal care of the mother and newborn” [24]. They also added 7.1% chlorhexidine digluconate to the 2013 Model List of Essential Medicines for Children. Although newborn mortality rates in Bangladesh are currently estimated at below 30/1,000, the Bangladesh Ministry of Health and Family Welfare (MOHFW) adopted a policy of universal single-dose cleansing of the cord stump on the day of birth with 7.1% chlorhexidine digluconate aqueous solution (CHX), regardless of place of delivery [16]. The national policy specified the product formulation, packaging, and application steps for chlorhexidine, as well as the required changes to provider training, health communications, and health information and logistics management systems in both public and private sectors (**Box 1**). The MOHFW also committed to scale up CHX by procuring CHX for the public health system and training maternal and newborn health (MNH) providers in public facilities in all 64 districts. The training included a 1-hour presentation on the evidence for CHX and videos demonstrating the correct method of application. There was then a 1-hour practical session, for participants to practice applying CHX to an anatomical model of a newborn. A local pharmaceutical company is supplying single-dose bottles of 7.1% CHX digluconate aqueous solution to the government at a cost of 24 taka (approximately US$0.30) per bottle. The MOHFW received technical and financial support for the scale up from the United States Agency for International Development (USAID) through its MaMoni Health Systems Strengthening (MaMoni HSS) Project. This implementation study was led by a team of researchers, newborn technical specialists and program monitoring staff supported by the MaMoni HSS project.

Box 1 **Summary of Bangladesh’s National Chlorhexidine Guidelines**

The 2014 national guideline, “Application of 7.1% Chlorhexidine Solution in the Newborn Umbilical Cord,” specifies the government’s implementation plan for providing chlorhexidine to all newborns, regardless of place of delivery. The key recommendations of the national guidelines are summarized below.

**Application guidelines:**

• A single application of chlorhexidine solution on the newborn umbilical cord stump should take place for all deliveries immediately after the cord is cut.

• For facility deliveries, the service provider should apply 7.1% chlorhexidine solution and inform the mother and family members.

• For home deliveries, a service provider or family member should apply 7.1% chlorhexidine solution.

• Irrespective of any condition, 7.1% chlorhexidine solution must be used on the umbilical cord stump. If chlorhexidine is not immediately applied after cutting the cord, it should be used anything within 48 hours of birth.

• No other substance should be applied to the newborn umbilical cord.

**Product guidelines:**

• 7.1% chlorhexidine digluconate solution, delivering 4% chlorhexidine digluconate, should be packaged in a purple 10 ml single use dropper bottle with pictorial user instructions.

**Capacity building of service providers:**

• Revised cord care guidelines and job aids will be developed and incorporated into all relevant training curriculum and standard operating procedures. All service providers should be oriented on the revised cord care guidelines.

**Social and behavioral change communication (SBCC) activities:**

• Pregnant women, their husbands, mothers-in-law, and other caregivers should receive counseling on the use of 7.1% chlorhexidine on the newborn umbilical cord stump.

• Health service providers at all levels, working in both public, private, and non-governmental organization facilities and in communities should conduct SBCC activities on essential newborn care, including chlorhexidine.

• The government should support mass media communications regarding 7.1% chlorhexidine.

**Supply chain management:**

• All antenatal care providers and community health workers should have sufficient supply of chlorhexidine dropper bottles to distribute to pregnant women in the third trimester. Pregnant women receiving the dropper bottle should be instructed to bring the bottle with them when they visit a facility for delivery.

• All facilities performing deliveries should have sufficient supply of 7.1% chlorhexidine dropper bottles.

• 7.1% chlorhexidine dropper bottles should be included in safe delivery kits.

• Pharmaceutical companies and social marketing networks should make 7.1% chlorhexidine dropper bottles available in the private market.

**Monitoring and health management information systems (HMIS):**

• The HMIS should be strengthened to capture 7.1% chlorhexidine application, including revisions to electronic reports and paper registers.

**Logistics management information systems (LMIS):**

• The LMIS should be strengthened to include reporting and recording of supply, storage and distribution of 7.1% chlorhexidine.

• Alert mechanisms should be included to avoid stock outs.

### Outcomes framework and measures

We developed an implementation outcomes framework for this study that adapts the RE-AIM measures [25] and incorporates ExpandNet’s conceptualization of scale up along the dimensions of institutionalization and expansion (**Table 1**). Under institutionalization, the outcomes framework includes qualitative measures of the adoption of CHX into policy and incorporation of CHX into plans, budgets, and health care delivery systems, informed by the Stages of Implementation Completion model [26]. Under expansion, the framework includes five quantitative measures addressing four RE-AIM constructs: reach, implementation, maintenance, and effectiveness. Reach assesses the extent of provider participation in CHX scale up, implementation assesses the extent to which CHX is delivered through government health services as intended, maintenance assesses the consistency of CHX service delivery over time, and effectiveness assesses the population-level coverage of CHX.

**Table 1:** Framework of implementation outcome measures

Dimension	Construct	Definition	Measure	Data Source
Institutionalization*	Adoption	Adoption of a policy supporting universal CHX†	Formal adoption of a policy supporting universal CHX	Qualitative interviews and document reviews
	Incorporation	Health systems/ organizational changes necessary to deliver CHX*^,^‡	Changes to work plans, budgets, logistics and reporting systems, training curriculum, etc., that incorporate CHX into health services	Qualitative interviews and document reviews
Expansion*	Reach	Provider participation in CHX scale up†	Number of providers (nurses and OB/midwives) who participated in training;	Program records
	Implementation	The extent to which CHX is delivered as intended†	Proportion of government facilities meeting readiness criteria for delivering CHX	Health Facility Survey
			Proportion of deliveries that receive CHX	HMIS data
	Effectiveness	Biologic or behavioral outcomes†	Population level coverage of CHX	Household survey in four districts
	Maintenance	The extent to which CHX becomes routine and a part of everyday culture†	Changes over time in population-level coverage of CHX	Household survey in four districts


CHX – chlorhexidine, HMIS – Health Management Information System

*Adapted from the ExpandNet/WHO framework for scaling up.

†Adapted from the RE-AIM framework.

‡Adapted from the Stages of Implementation Completion Framework.

### Data sources and analysis

This study draws on several routine and secondary quantitative data sources, for measures of expansion indicators. Additionally, we conducted qualitative interviews, focus group discussions, and content analysis of program documents to assess institutionalization processes and outcomes. Data collection and analysis methods are described below by data source.

#### Program documents (Adoption and Incorporation)

We collected and reviewed over 20 program documents, including policy documents (eg, national guidelines, government orders), presentations, and meeting minutes. We performed content analysis to identify key milestones in adoption and incorporation and the dates when they were achieved.

#### Qualitative interviews with national stakeholders (Adoption and Incorporation)

We also conducted qualitative interviews with stakeholders at the national and district level to collect data on processes used to achieve scale up milestones. An initial list of stakeholders was developed by members of the study team, with input from the national scale up team, and expanded using a snowball approach with early interview participants. A total of 25 informants participated in individual or group interviews, including program managers for the government (MOHFW) and partner organizations (eg, MaMoni HSS, the national post-graduate medical university, UNICEF, professional associations, members of national newborn technical working committees, nongovernmental providers, pharmaceutical company executives, and program trainers). Members of the study team conducted interviews between December 2016 and March 2018. A team of two research staff conducted each interview; one researcher led the interview while the other took detailed hand written notes. All interviews were audio recorded with the informants’ permission. Following each interview, the team carried out an immediate debriefing session with one of the study investigators. Expanded notes were written within 1 day of each interview or FGD, with reference to the audio recordings as needed [27]. The qualitative data collection for this study was reviewed and judged to be exempt by the JHSPH IRB under Department of Health and Human Services Regulation 45 CFR 46.101(b), Category (2) (Protocol #6950).

Analysis of qualitative data was guided by the Framework approach [28] and rapid ethnographic methods for programmatic qualitative research [29]. Following familiarization and open coding of one-quarter of the transcripts, we developed a coding framework including both inductive and deductive themes categorized according to the five domains in the Consolidated Framework for Implementation Research [30], which was refined with feedback from multiple authors. Notes were coded in Dedoose [31] and coded passages were summarized in charts by theme and respondent [28]. This paper reports the qualitative findings describing institutionalization and expansion processes. Initial findings and interpretations were shared and refined with feedback from all authors.

#### Training records (Reach)

Records of the dates, locations, and number of providers trained were maintained in Microsoft Excel (Microsoft Inc, Seattle, WA, USA) by the National Newborn Health Cell, located in the program unit responsible for newborn interventions within the MOHFW. These records were supplied by local partner organizations contracted to conduct the trainings. We tallied the total number of participants across trainings and by position title in Excel.

#### Facility readiness assessment (Implementation)

Following the training of health workers on CHX, the MaMoni HSS project also contracted Bangabandhu Sheikh Mujib Medical University and a national non-government organization, Partners in Health Development, to conduct a nationwide assessment of health facility readiness to deliver a small set of priority newborn interventions, including CHX. The lowest level facilities included were Union Health & Family Welfare Centers, and the highest-level facilities included were district hospitals. Retired government doctors visited every facility designated to provide skilled delivery and completed a standard checklist that included assessment of the following readiness data related to CHX: CHX training status of all MNH providers at the facility, presence of the newborn job aid addressing CHX, and the stock of the CHX commodity. The data collection was performed at 4479 facilities between October 2016 and December 2017. We calculated the proportion of facilities meeting three readiness criteria: 1) 75% or more of their MNH providers trained in CHX; 2) presence of at least one copy of the job aid; and, 3) CHX in stock at the facility. We also calculated a composite indicator of the proportion of facilities meeting all three criteria. Analyses were performed in Stata 15 (StataCorp LP, College Station, Texas, USA) [32].

#### Health management information system data (Implementation)

Under the Bangladesh public health system, maternal health services are provided by facilities operated under two separate directorates – the Directorate General for Health Services (DGHS) and the Directorate General for Family Planning (DGFP) [33]. Obstetric providers in both directorates record each birth, the outcome of the birth (live or stillborn) and the care provided to live newborns, including CHX cleansing of the umbilical cord stump in paper-based registers (Maternal and Newborn Health registers in DGFP facilities and Emergency Obstetric Care registers in DGHS facilities). These data are aggregated into monthly facility reports and entered into the District Health Information System (DHIS2) for DGHS and the Management Information System (MIS) for DGFP. In January 2018, we accessed the web-based DHIS2 and MIS and downloaded monthly aggregate values of live births and CHX applications for the 12-month period between December 2016 and November 2017. We merged these data sets and calculated the number and proportion of live newborns that received CHX.

#### Household survey (Effectiveness and Maintenance)

In low-income settings, household surveys are often required to estimate population-level coverage of interventions. The Demographic and Health Survey (DHS), the most common household survey addressing MNH in low-income countries, was most recently conducted in Bangladesh in 2014 and again in late 2017 through early 2018, with results and data set not expected to be publicly available until mid-2019. In order to achieve more frequent and rapid estimates of the coverage of priority MNH interventions, the MaMoni HSS project partner, icddr,b, conducted rolling household surveys in four districts with the largest project investments (Habiganj, Jhalokathi, Lakshmipur, and Noakhali). The surveys were conducted under a separate research protocol and data were made available to our team for secondary analysis. The survey methods were reviewed and approved by the Institutional Review Boards of the Johns Hopkins Bloomberg School of Public Health (Protocol #5931) and icddr,b (Protocol #15-122).

Each survey followed a multi-stage cluster sampling approach to achieve annual estimates of priority indicators for each of 23 upazilas (sub-districts) served by the MaMoni HSS program. Within each upazila, between 8 and 27 clusters were randomly sampled with probability proportional to size from the 2011 national census listing. The sampling plan required sampling clusters from every union (the lowest administrative unit), in the 23 upazilas. Each cluster consisted of a population of 300 to 350 households, from which 4 women who had delivered a live baby in the previous 6 months would be interviewed. The number of clusters sampled within each union was determined by the population of the upazila and the number of unions it contained. A survey team visited each sampled cluster and then randomly selected an index case from the register of all currently married woman of reproductive age maintained by the Family Welfare Assistant (FWA) in each union. FWAs are community-based health workers employed by the MOHFW and are responsible for maintaining a register of all couples of reproductive age within their defined catchment area and visiting each couple every two months.

Within each sampled cluster, the survey team visited the index household to screen the household for an eligible woman and administer the survey, if an eligible woman consented. The survey team then proceeded to each next nearest household in the cluster, in a clockwise direction, until a total of 4 eligible women were identified and agreed to participate in the survey. Using standard questionnaires, interviewers asked mothers to recall the birth outcome (live or stillborn), location of delivery, and the care the newborn received, including CHX. We calculated the proportion of live births that received CHX for six consecutive rounds of the survey, covering the period from March 2014 through August 2017. Mothers who reported that they did not know whether their baby received CHX were included among those who did not receive CHX in analysis.

## RESULTS

### Implementation processes and outcomes

#### Adoption and Incorporation

**Table 2** details adoption and incorporation milestones for the scale up of cleansing of the umbilical cord stump with 7.1% CHX in Bangladesh. Newborn health policy in Bangladesh’s public health system is led by the National Core Committee for Newborn Health, while the technical and implementation aspects are led by a National Technical Working Committee for Newborn Health (NTWC), a group convened by the MOHFW, and composed of stakeholders from government, academia, and nongovernmental organizations. The process of adopting a policy in support of CHX spanned three years, starting in 2011, when officials from the Government of Bangladesh and the NTWC attended a meeting convened by USAID to disseminate results from three CHX trials. Following a national consultation workshop in 2012, organized by the NTWC and supported by the Saving Newborn Lives Program of Save the Children, and discussion of CHX at several NTWC meetings, the NTWC recommended replacing the national policy of dry cord care with universal cleansing of the umbilical cord with CHX, as did four professional organizations.

**Table 2:** Adoption and incorporation milestones

Domain	Milestone	Date
**Adoption**		
Policy	Regional dissemination workshop on CHX attended by Government of Bangladesh officials, Katmandu, Nepal	September, 2011
Policy	National consultation workshop on CHX, Dhaka, Bangladesh	January, 2012
Policy	National Technical Working Committee Endorses CHX for National Policy	May, 2013
Policy	Professional societies also endorse CHX policy adoption	June, 2013
Policy	“A Promise Renewed” published by Government of Bangladesh, prioritizing 4 newborn health interventions, including CHX	July, 2013
Policy	National guidelines for CHX developed and finalized by National Technical Working Committee	July, 2014
Policy/Capacity building	Comprehensive Newborn Care Package training curriculum with CHX finalized	December, 2014
**Incorporation**		
Plans & Budgets	Operational plans and budget of the DGHS and DGF, under the 3rd Health, Population, and Nutrition Sector Development Program 2011-2016, revised to incorporate CHX	2015
Capacity building	CHX included in pre-service training curriculum	January, 2015
Procurement & Logistics	CHX included in online logistics management system	January, 2015
Procurement & Logistics	Production of CHX by local company begins	March, 2015
Procurement & Logistics	First government procurement of CHX (600 000 bottles by DGHS)	May, 2015
Procurement & Logistics	Commercial production and distribution by local company begins	June, 2015
Capacity Building	Training of providers begins	July, 2015
Procurement & Logistics	Second government procurement of CHX (154 000 bottles by DGFP)	December, 2015
Procurement & Logistics	First distribution of CHX bottles to government health facilities	January, 2016
Capacity Building	Training of providers completed	September, 2016
Monitoring	New Emergency Obstetric and Newborn Care register use begins, including CHX provision indicator	January, 2017
Plans & Budgets	Approval of the 4th Health, Population, and Nutrition Sector Development Program 2017-2021, including CHX plans and procurement budget for both DGHS and DGFP	March, 2017
Capacity Building	Refresher training on essential newborn care package, including CHX for new providers	August, 2017 – March, 2018
Behavior Change	National newborn communications campaign launched	November, 2017

CHX – chlorhexidine, DGHS – Directorate General Health Services, DGFP – Directorate General of Family Planning

The first official government policy adopting CHX in Bangladesh was the declaration “Ending Preventable Child Deaths by 2035: Bangladesh Call for Action,” published in July 2013. Country governments were encouraged to develop such strategy declarations under the “A Promise Renewed” initiative organized by USAID and UNICEF. A pilot implementation was conducted with facility- and community-based government health workers in a limited area by the MaMoni HSS project, and the operational recommendations from this pilot were incorporated into the NTWC’s detailed national guidelines for CHX in July 2014 (Panel 1), and a provider training curriculum, developed by Saving Newborn Lives, was finalized in December 2014.

Following the policy adoption, completion of the incorporation milestones required for newborns to begin receiving CHX at government health facilities took an additional 2 years. The ongoing operational plans and budgets of DGHS and DGFP were revised to accommodate CHX procurement in 2015, and an initial procurement was made by DGHS in May 2015. At the same time, an intensive initial capacity building effort to train MNH providers at all public health facilities started in July 2015 and was completed in September 2016.

**Figure 1:**
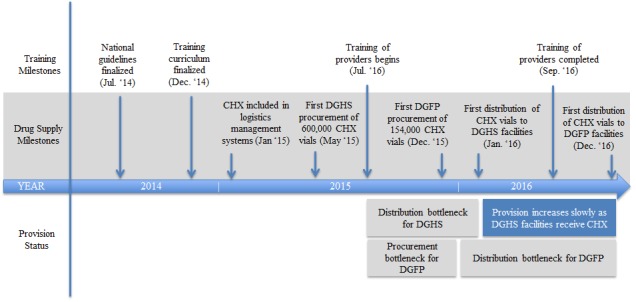
Timeline of selected incorporation milestones and their impact on provision of chlorhexidine. CHX – chlorhexidine, DGHS – Directorate General of Health Service, DGFP – Directorate General of Family Planning.

Although providers in many districts were trained by the end of 2015, and a quantity of commodities had been procured, delays with institutionalization within the supply distribution system were a major bottleneck for scale up. While the MOHFW’s first procurement of 600 000 bottles of CHX for DGHS occurred in May 2015, this was placed in the central medical stores and no bottles were disbursed to DGHS health facilities until January 2016 (**Figure 1**). DGFP procured 154 000 bottles in December 2015, and these were held in the central warehouse, awaiting incorporation of CHX into the logistics management information system before they could be distributed to facilities, which occurred in December 2016. As a result, many providers were trained six months to a year before any CHX was available at their facilities. A proportion of bottle in first batches of CHX that were purchased a year before distribution ended up expiring before they could be used by facilities. At the same time, the local manufacturer, ACI Pharmaceuticals, made the product commercially available through its retails network across all 64 districts of the country beginning in June 2015. The private and NGO sector facilities were able to procure the product from the commercial market and/or advise the families to buy it at the time of delivery.

#### Reach

A half-day in-service training for CHX was initially rolled out in a phased manner by district, over a 14-month period from July 2015 to September 2016. The MOHFW contracted four local partner organizations to conducted training of all public facility MNH providers in each district, and also hired retired physicians to monitor the trainings. According to the training data maintained by MOHFW, a total of 80 780 providers were trained during the initial training between July 2015 and September 2016. The trainees included Family Welfare Assistants (22.6%), Health Assistants (18.8%), Community Healthcare Providers (15.4%), medical officers (12.7%) and nurses (9.9%). Gender data were collected for 95% of participants, indicating that approximately 57% were female and 38% were male (Table S1 in **Online Supplementary Document**). The same half-day training was also provided to approximately 425 private and NGO providers who participated in 7 regional advocacy and train-the-trainer meetings.

#### Implementation

The facility readiness assessment provided data on the extent to which 4479 public health facilities were prepared to deliver the new CHX intervention as intended. Among all facilities, 3293 (74%) had at least three-quarters of the MNH providers in the facility trained in CHX cord cleansing. The job aid used for counseling on essential newborn care, “*Saf Kotha*”, was present in 3365 (75%) facilities. While three-quarters of facilities were ready with trained providers and a job aid, only 2069 (46%) of facilities had a supply of CHX on the day of the assessment. These results confirm the observations of interview participants, that even after distribution of CHX started, supply was irregular or insufficient to many facilities. When combining all three indicators into a composite readiness score, 1510 (34%) of facilities met all three of the above criteria.

**Figure 2:**
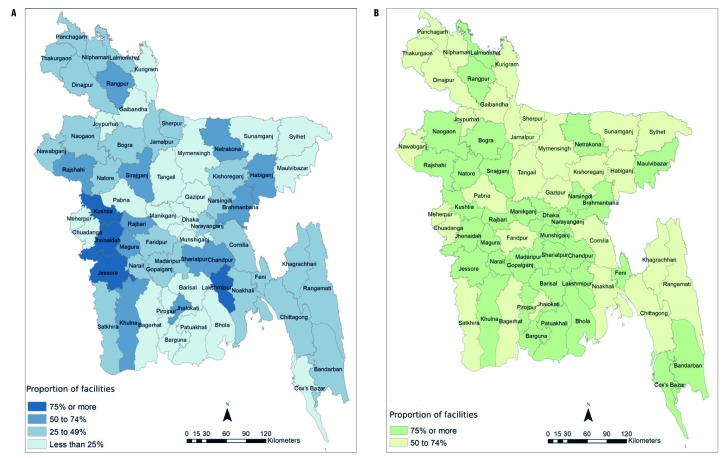
Proportion of government facilities that perform deliveries meeting composite readiness and training criteria, by district. **Panel A.** Proportion of district facilities meeting three readiness criteria (75% training, job aid, and chlorhexidine in stock). **Panel B.** Proportion of district facilities having at least 75% of providers trained in the Emergency Obstetric and Newborn Care package, including chlorhexidine cord cleansing.

**Figure 2** provides a map illustrating the proportion of facilities ready to provide CHX cord cleansing in each district, by the composite readiness criteria (75% of MNH providers trained, job aid available, and CHX in supply) and by the training readiness criteria only. When considering only the training criteria, 75% or more of facilities in 35 districts met the training readiness criteria (district range: 51% to 95% of facilities). In contrast, only 4 of 64 districts had 75% or more of their facilities meeting all three composite criteria, while in 22 districts fewer than 25% of facilities met the three criteria (district range: 0 to 82%). It should be noted that due to the large scope of the assessment, the data collection process took approximately 14 months, and districts were visited at different times during this period.

**Figure 3:**
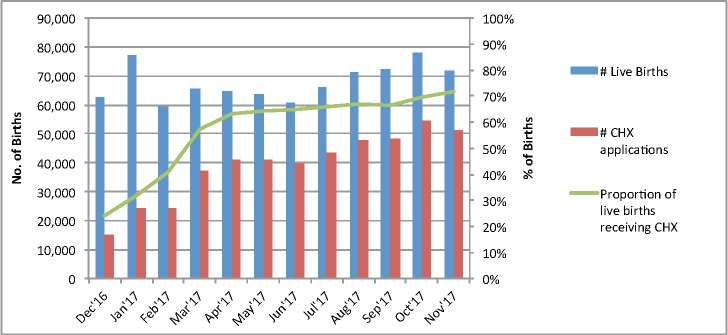
Number and proportion of live births receiving chlorhexidine at government facilities between December 2016 and November 2017.

Our second measure of implementation was the proportion of deliveries at public facilities that received CHX according to HMIS records. As demonstrated in **Figure 3**, there was a rapid increase in newborns receiving CHX in the first months of 2017. In December 2016 – the first month that CHX was tracked in the HMIS – 15 059 (24%) of the 62 672 reported live births received CHX. This proportion increased an average of approximately 10 percentage-points per month between January and April 2017, followed by continued gradual increases around 1 percentage-point per month maintained from May through November 2017. In the month of November 2017, 72% of the 71 704 live births received CHX. Across the 12-month period, a total of 468 249 live newborns (58%) were provided with CHX cleansing of the umbilical cord stump.

#### Effectiveness and Maintenance

We measured population-level effectiveness and maintenance, defined as the coverage of CHX among live births and changes in coverage over time, through the MaMoni HSS project four-district household survey. A total of 39 585 women participated in six rounds of the survey from September 2014 through August 2017, with an average of ~ 6600 women who recently delivered a live baby included per survey round (**Table 3**). In each survey, more than half of the women delivered at home, and the percentage that delivered in a public facility ranged from 11 to 18%. The percentage of mothers delivering at a public facility who reported that their baby received CHX started a little over 2% in rounds 1 and 2, and increased significantly in each subsequent round, finally reaching 57.7% (95% CI = 55.1, 60.2) in round 6. Population-based coverage of CHX for all live births, regardless of place of delivery, also increased in each survey after round 2, reaching 33% in round 6 (95% CI = 31.9, 34). In each round, the coverage of CHX among all live births receiving CHX was about half the coverage among live births in public facilities. In the final survey, 57.7% (95% CI = 55.1, 60.2) of babies born in public sector hospitals received CHX, compared with 41.1% (95% CI = 39, 43.2) born in other facilities and 21.3% (95% CI = 20.1, 22.5) of home births (Table S2 in **Online Supplementary Document**).

**Table 3:** Population-level coverage of chlorhexidine among live births in four districts

Survey round (dates)	Sample of women with a recent live birth (% who delivered in a public facility)	Live births in public facilities		All live births	
**Proportion receiving CHX**	**% change from previous survey**	**Proportion receiving CHX**	**% change from previous survey**
1 (9/14-2/15)	6351 (11%)	2.5 (1.5, 3.9)	N/A	2.5 (2.2, 3)	N/A
2 (2/15-8/15)	6158 (13%)	2.1 (1.2, 3.4)	-16%	1.9 (1.6, 2.3)	-24%
3 (9/15-2/16)	6378 (14%)	8.7 (7, 10.8)	314%**	4.3 (3.8, 4.8)	126%*
4 (3/16-8/16)	6157 (16%)	20.6 (18.2, 23.3)	137%**	9 (8.3, 9.8)	109%**
5 (9/16-2/17)	6379 (17%)	40.2 (37.3, 43.2)	95%**	19.3 (18.3, 20.3)	114%**
6 (3/17-8/17)	8162 (18%)	57.7 (55.1, 60.2)	44%**	33 (31.9, 34)	71%**

CHX – chlorhexidine

Significance test for difference in proportions with previous year: *P < 0.05; ** *P* < 0.01.

## DISCUSSION

This study leveraged multiple data sources to evaluate implementation outcomes for a nationwide scale up of chlorhexidine of the umbilical cord cleansing in public health facilities in Bangladesh, which replaced the previous national policy of dry cord care. While CHX was initially endorsed by the MOHFW in July 2013, the process of institutionalizing all changes into the health system that were required to enable provision of CHX to newborns delivered at public facilities took an additional 2.5 years. The scale up efforts achieved high reach of providers, training over 80 000 health workers representing all 64 districts of the country. Implementation improved throughout 2017, with the proportion of newborns that received CHX in public facilities passing 70%. The MOHFW and local manufacturer also continued to actively engage the private and non-governmental organizations to make the product available in the market and to promote its use in births happening at home and private/ NGO facilities. In the four districts with available survey data, population-level coverage of CHX continuously increased between 2015 and 2017, eventually reaching an estimated one-third of all newborns.

The findings from this study highlight the importance of institutionalization milestones for successful scale up. In order to ensure availability of CHX in Bangladesh, changes needed to be made to budgets to allow procurement and to the logistics and distribution systems for two separate directorates. Incorporating CHX into the distribution systems, in particular, was a more difficult and time-consuming process than planned for, and this gap in institutionalization delayed expansion and service delivery to many newborns. Bangladesh’s experience suggests that scale up efforts should involve early needs assessments and planning for institutionalizing new drugs and commodities into the supply chain. Even after institutionalization, maintaining the supply of a new drug and commodity depends on the functioning of the existing logistics systems, unless the scale up includes investments in strengthening the existing system or establishing parallel systems, which are considered to have negative effects on efficiency and sustainability [34].

To date the scale up of CHX has primarily taken place in public facilities, yet only 14% of deliveries take place in public facilities, while 29% take place in private facilities and 53% take place at home [23]. Although the national guidelines recommend distribution of CHX during ANC and by community health workers for home births, these delivery channels have not been implemented in the public sector to date, in part due to inadequate procurement of CHX for wider distribution. Outside the public sector, other large NGO providers are adopting CHX much later than the government, citing cost as the primary barrier. For example, two health NGOs, Brac and the Social Marketing Company, sell popular kits for clean home deliveries that cost approximately US$1. Adding CHX at the current market rate would double the cost of these kits. In the private sector, where the majority of facility births take place, there are few regulatory mechanisms and little data about the quality of care provided. Sales records for CHX from private outlets, provided by the local manufacturer of CHX, demonstrate continued increases in purchases of CHX, averaging ~ 30 000 bottles per month during 2017 (Figure S1 in **Online Supplementary Document**). Further investigations would be needed to understand what factors are contributing to growth in private sales.

To guide the assessment of implementation outcomes for this study, we adapted the RE-AIM framework [25], incorporating concepts from the WHO/ExpandNet scale up model [10] and the Stages of Implementation Completion [26]. The concept that we label “incorporation” encompasses necessary institutionalization changes to work plans, budgets, and information and management systems, which are prerequisites to meeting later implementation outcomes such as reach and effectiveness. The example of commodity procurement and distribution bottlenecks during the scale up of CHX in Bangladesh demonstrates that these institutionalization outcomes are critical in achieving scale up. However, common implementation frameworks tend to underemphasize these milestones, or to consider them as a part of health systems contextual factors rather than implementation outcomes. For example, both RE-AIM and Proctor’s “taxonomy of implementation outcomes” [35] consider adoption as an initial decision or intention to implement, but neglect the institutionalization steps between the adoption decision and service provision. Specific to low-income countries, Hanson and colleagues developed a typology of constraints to scaling up, including drug and supply systems, as well as planning and management issues and overall governance [12]. Similarly, the “Framework for Going to Scale,” developed by the Institute for Healthcare Improvement, includes support systems for going to scale, but does not include changes to these systems as a part of phases of scale up [8]. Further methodological development is needed to enumerate and define common institutionalization outcomes and incorporate them into frameworks used by evaluators and planners of scale up in low-income countries.

In order to assess implementation outcomes on a national scale, this study relied on several routine data sources, including HMIS data, program records, and a facility readiness assessment conducted for program purposes. We were not able to assess the quality of data from these sources, although HMIS data in particular is known to have completeness and accuracy problems in low-income country contexts [36]. The only data available on population-level coverage of CHX is limited to a household survey in 4 of 64 districts, and these are districts that received additional health systems strengthening support from the MaMoni HSS project. The population coverage of CHX in the four surveyed districts is likely somewhat higher than districts without development partner investments in health systems strengthening. Household surveys rely on maternal recall of newborn care to measure CHX coverage and some mothers may not know whether their child received CHX at birth, particularly mothers who delivered at a facility where the provider may or may not explain the care provided to the newborn. While there is no validation data specifically on CHX, maternal recall of other newborn care indicators, such as breastfeeding within one hour, immediate drying, and skin-to-skin care, have mixed sensitivity and specificity [37]. Our analysis may be an underestimate of CHX coverage among facility deliveries, as one-fifth to one-third of women delivering at a facility reported that they did not know whether their baby received CHX in each survey round. A separate limitation of the survey methods is sampling the index households for each cluster from the registers of community-based FWAs. It is possible that some geographic areas are excluded if the FWA registers are not complete listings of women of reproductive age in their catchment areas and that excluded areas are less likely to access care. Despite the limitations of the household survey methods, these data provide important evidence of increasing CHX coverage for the larger population, even among women who do not deliver in public facilities.

## CONCLUSIONS

The scale up of CHX in Bangladesh achieved national implementation in the public health sector. To achieve a greater public health impact, the scale up effort must expand to reach deliveries that take place at home and in the private sector. Institutionalization milestones, such as changes to supply logistics systems, were barriers that had to be addressed before expansion of service provision was achieved. Greater attention to institutionalization processes and outcomes in implementation frameworks and studies would advance our understanding of how to achieve successful scale up, particularly in low-income country contexts.

## Additional material

Online Supplementary Document
