# Supplementary material for: Implementation outcomes of the national scale up of chlorhexidine cord cleansing in Bangladesh’s public health system
Source: J Glob Health. 2019 Sep 17;9(2):020410. doi: 10.7189/jogh.09.020410 (PMC6793170; doi:10.7189/jogh.09.020410)
Supplement: Online Supplementary Document [file jogh-09-020410-s001.pdf]

Table S1. Characteristics of participants in the initial CHX scale up training.

Characteristic	Number	% of Total
**Training participants by cadre**		
Community Healthcare Provider (CHCP)	12,412	15.4%
Family Welfare Assistant (FWA)	18,224	22.6%
Family Welfare Visitor (FWV)	4,116	5.1%
Health Assistant	15,191	18.8%
Medical Officer	10,291	12.7%
Surgeon/Assistant Surgeon	844	1%
Nurse	8,033	9.9%
Other	11,669	14.4%
TOTAL	80,780	100%
**Training participants by gender (data missing for ~5%)**		
Male	30,964	38.3%
Female	45,906	56.8%
Total participants with gender data (% of all participants)	76,941	95.2%

Table S2. Changes in population-based coverage of chlorhexidine and other cord care in 4 districts of Bangladesh.

Indicator	Round 1 (9/14 – 2/15)	Round 2 (2/15 – 8/15)	Round 3 (9/15 – 2/16)	Round 4 (3/16 – 8/16)	Round 5 (9/16 – 2/17)	Round 6 (3/17 – 8/17)
**All Births**						
Chlorhexidine	2.5 (2.2,3)	1.9 (1.6,2.3)	4.3 (3.8,4.8)	9 (8.3,9.8)	19.3 (18.3,20.3)	32.9 (31.9,34)
Dry cord care/nothing	50.1 (48.8,51.3)	54.1 (52.9,55.4)	51.9 (50.7,53.1)	38.8 (37.5,40)	31.6 (30.5,32.8)	21.3 (20.4,22.2)
Other substance	38.3 (37.1,39.5)	30.9 (29.7,32)	31.7 (30.6,32.9)	39.3 (38.1,40.6)	32.5 (31.3,33.6)	35.5 (34.5,36.6)
Don't know	9.1 (8.4,9.8)	13.1 (12.3,14)	12.1 (11.3,12.9)	12.9 (12,13.7)	16.6 (15.7,17.6)	10.2 (9.6,10.9)
**Home Births**						
Chlorhexidine	2.1 (1.7,2.5)	1.7 (1.3,2.2)	3.8 (3.3,4.5)	5.7 (5,6.5)	14 (12.9,15.2)	21.3 (20.1,22.5)
Dry cord care/nothing	53.3 (51.8,54.8)	59.4 (57.8,60.9)	57 (55.5,58.5)	42.9 (41.3,44.5)	38.7 (37.2,40.3)	26.6 (25.3,27.9)
Other substance	40.6 (39.2,42.1)	33.8 (32.4,35.3)	34.7 (33.2,36.1)	48.3 (46.7,49.9)	43.6 (42,45.2)	49.6 (48.1,51)
Don't know	4.1 (3.5,4.7)	5.1 (4.4,5.8)	4.5 (3.9,5.2)	3.1 (2.6,3.8)	3.7 (3.1,4.3)	2.6 (2.1,3.1)
**Public Sector Facility Births**						
Chlorhexidine	2.5 (1.5,3.9)	2.1 (1.2,3.4)	8.7 (7,10.8)	20.6 (18.2,23.3)	40.2 (37.3,43.2)	57.7 (55.1,60.2)
Dry cord care/nothing	46.8 (43.1,50.5)	51.2 (47.6,54.8)	43.9 (40.7,47.2)	32.7 (29.8,35.7)	18 (15.8,20.4)	13.2 (11.5,15)
Other substance	27.6 (24.4,31)	15.3 (12.9,18.1)	20.8 (18.2,23.5)	20.7 (18.3,23.4)	13 (11.1,15.1)	13.9 (12.2,15.8)
Don't know	23.1 (20,26.3)	31.4 (28.2,34.8)	26.6 (23.7,29.5)	26 (23.3,28.8)	28.8 (26.1,31.6)	15.3 (13.5,17.2)
**Other Facility Births**						
Chlorhexidine	4.3 (3.3,5.7)	2.4 (1.6,3.3)	2.8 (2,3.8)	9.6 (8.1,11.2)	17.1 (15.2,19)	41.1 (39,43.2)
Dry cord care/nothing	40.2 (37.4,43)	39.5 (36.8,42.2)	41.6 (38.9,44.3)	32.6 (30.2,35.1)	24.2 (22.1,26.4)	15.4 (13.9,17)
Other substance	36.2 (33.4,39)	30.7 (28.2,33.3)	30.1 (27.6,32.6)	29.3 (27,31.7)	19.4 (17.4,21.5)	20.2 (18.5,22)
Don't know	19.3 (17.1,21.6)	27.5 (25.1,30)	25.6 (23.3,28)	28.5 (26.2,30.9)	39.4 (36.9,41.9)	23.3 (21.6,25.2)

Notes: Survey covers Habiganj, Jhalokathi, Lakshmipur, and Noakhali districts.

Figure S1. Sales of Chlorhexidine bottles by ACI Pharmaceuticals between January 2015 and December 2017.

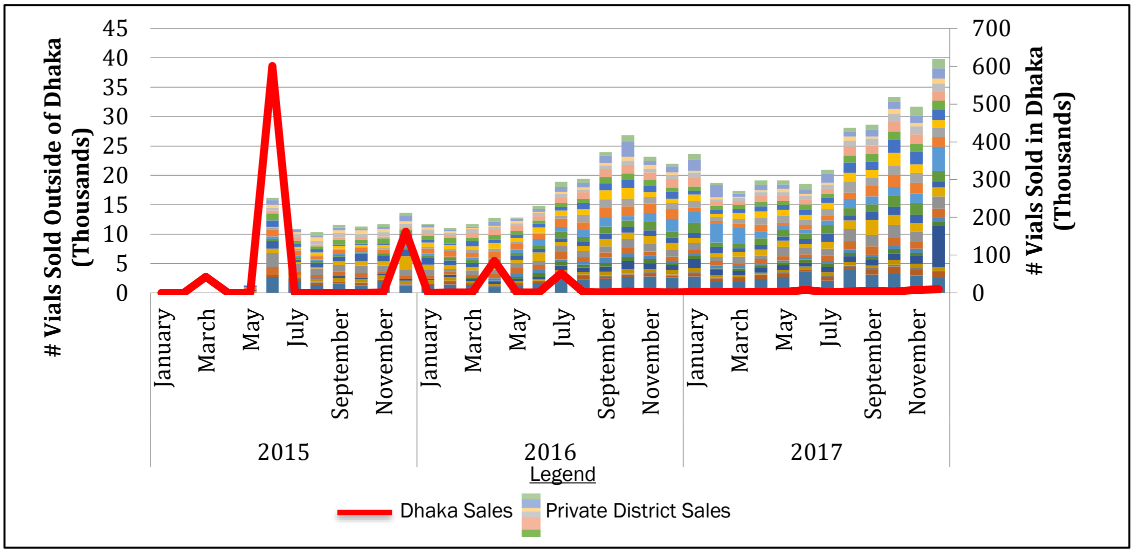

**Notes: The line graph depicts CHX sales for Dhaka, including sales to the Government of Bangladesh for national distribution. The histogram bars reflect private sector sales in districts, with each color in the stacked bars showing the total sales for an individual district.*
